# Local Demand, Quality of Place, and Urban Tourism Competitiveness

**DOI:** 10.3389/fpsyg.2021.817805

**Published:** 2022-02-15

**Authors:** Jin Weng, Jiaying Xiao, Larry Yu

**Affiliations:** ^1^Department of Tourism, Fudan University, Shanghai, China; ^2^Department of Management, School of Business, The George Washington University, Washington, DC, United States

**Keywords:** quality of place, urban tourism competitiveness, local demand, global cities, mediating effect

## Abstract

Most studies on tourism destination competitiveness examined the direct relationships of destination attributes and destination competitiveness. Few studies explored the intervening mechanisms between destination attributes and competitiveness. This study selected cities above the “alpha” level in the Globalization and World Cities Research Network rankings as samples to examine the relationship between local demand and urban tourism competitiveness mediated by the quality of place. Results showed that the relationship between urban wealth and tourist arrivals was completely mediated by the quality of place, while the mediating effect was not significant in the relationship between urban wealth and per capita tourism spending, though per capita tourism spending was directly and positively associated with urban wealth. In other words, richer cities had a higher quality of place, and a higher quality of place attracted more tourists but did not increase tourism per capita spending. Furthermore, the study found that there were two opposite influence paths of city size on urban tourism competitiveness. Although urban tourism competitiveness was directly and positively affected by population, the population had a significant negative effect on urban tourism competitiveness mediated by the quality of place. Through the quality of place, we can be aware of the indirect negative effect suppressed by the direct positive effect. This study revealed that the quality of place helped to better understand the competitiveness of urban tourism.

## Introduction

Theorizing the framework of destination competitiveness has been the main focus of tourism destination competitiveness (TDC) studies in the last two decades (Crouch and Ritchie, 1999; Dwyer et al., 2004; Enright and Newton, 2004, 2005; Assaker et al., 2013; Kruger and Heath, 2013; Cvelbar et al., 2016; Mendola and Volo, 2017; Fernández et al., 2020). Different conceptual frameworks and methodological approaches have been used for defining and measuring destination competitiveness. One strand of literature focused on core tourism resources and attractions and institutional arrangements such as destination organization management, destination policy and planning, environmental factors, and tourism industry attributes. These studies aimed to develop and refine comprehensive models for determining composite indicators to measure destination competitiveness using multidimensional variables at different territorial scales (Mendola and Volo, 2017), e.g., urban destination competitiveness (Enright and Newton, 2004, 2005; Cibinskiene and Snieskiene, 2015; Evren and Kozak, 2018), and national destination competitiveness (Dwyer et al., 2004; Assaker et al., 2013; Lee, 2015; Cvelbar et al., 2016; Fernández et al., 2020). TDC is, therefore, a broad term that encompasses the study of the competitiveness of tourism destinations of different spatial scales and different characteristics, such as national, regional and local, or urban vs. rural destinations. This study focuses on urban tourism competitiveness which is a critical aspect of TDC research. Other studies examined different determinants of TDC (Cvelbar et al., 2016), namely, smart applications of technology in destination management (Boes et al., 2016; Koo et al., 2016); macroeconomy influencing TDC through public goods such as the environment and infrastructure (Mihalič, 2000; Assaker et al., 2013); the effect of sustainable indicators in TDC (Cucculelli and Goffi, 2016); policy and planning factors influencing TDC (Zehrer and Hallmann, 2015); and the impact of globalization on TDC (Ivanov and Webster, 2013). These studies further complemented the comprehensive and multidimensional studies by providing nuanced insights into tourism destinations’ competitiveness of different scales and characters.

However, Novais et al. (2018) noted the limitations of extant studies in conceptualizing TDC without recognizing the perceptions of the stakeholders and argued that the concept of destination competitiveness needs to consider three critical orientation focuses: perception, performance, and long-term process. Perception deals with the demand that examines the relationship between consumers and destinations, and visitor experience from visiting the destination. The performance focuses on the product supply of the destination and the measurable performances. The long-term process offers a holistic perspective on the supply and demand in the destination as a system and its impact on the wellbeing of the destination. This new conception of TDC thus incorporated the different perceptions of destination stakeholders: destination governance, service providers, local residents and tourists, and examined the balance between tourists and destination, tourists and local residents, and local destination and local residents.

Moreover, most extant studies on TDC have focused on country destinations (Dwyer et al., 2004; Assaker et al., 2013; Lee, 2015; Cvelbar et al., 2016; Fernández et al., 2020). Few studies explored urban TDC, except for the studies on city tourism competitiveness in Asia (Enright and Newton, 2004, 2005) and the study by Cibinskiene and Snieskiene (2015) on determining weight coefficients by expert analysis of internal and external environmental factors. Both studies examined a wide array of factors contributing to the competitiveness of urban tourism development. However, most studies have examined the direct impact of the various resource, economic, social, and institutional factors on destination competitiveness. Only one research studied the relationships of the national economy on country destination competitiveness mediated by physical environment and infrastructure (Assaker et al., 2013). This study revealed a positive and indirect mediating effect of physical environment and infrastructure between the national economy and country destination competitiveness, while physical environment and infrastructure had a direct effect on destination competitiveness. This study provided a novel, nuanced insight into TDC research by examining the intervening mechanisms underlying the national economy and the ranking of TDC.

According to Porter (1990), the ability to provide products and services is affected by many factors, such as the factor conditions of the destination, local demand conditions, related and supporting industries, and market structure. Diamond model by Porter emphasizes the importance of local demand conditions, but in the traditional research on TDC, local demand has been ignored. Based on the results of questionnaire surveys, Enright and Newton (2004, 2005) believe that local demand is not the main factor of TDC. One possible explanation is that the impact of local demand on destination tourism development is indirect, and it is not easy to be directly observed by the observers, so the observers tend to underestimate the impact of local demand on destination tourism development.

In this study, we introduced the quality of place as a mediating variable to determine the impact of the local population and income level on TDC. The concept of the quality of place proposed by Florida (2002) has provided a new perspective to understand TDC. The quality of place is the key factor of urban competitiveness, which refers to “certain characteristics of the day-to-day urban environment” (Trip, 2007, p. 502) and is represented by the type, quantity, and quality of amenities (Yang, 2019). Cities with the high quality of place tend to improve the quality of life of residents, a subjective feeling of well-being or satisfaction with the place, and, thus, attract the “creative class,” defined by Florida as those who have a “supercreative core” or are “creative professionals” that help attract talent-oriented corporations and drive urban economies, especially the advanced service economies (Florida, 2002). The lifestyle of the creative class, in turn, reshapes the unique quality of place, which can also be regarded as a “competitive asset for attracting tourists” (Reilly and Renski, 2008, p. 17). Experiencing the lifestyle of the creative class has become an important tourism product. The quality of place initially provided for long-term residents can also be shared with tourists. Therefore, the quality of place plays an important role in the development of urban tourism. However, there is still limited discussion on the relationship between the quality of place and TDC in the extant literature.

This study selected cities above the “alpha” level in the Globalization and World Cities Research Network rankings as samples and used the index system provided by Trip (2007) to measure the quality of place of 50 global cities. Then, the quality of place was introduced as a mediator variable to examine the relationship between local demand, the quality of place, and TDC. The novelty of this research is to apply the mediation model by Hayes (2013) to test and uncover the direct, indirect, and even potential suppressing effects between local demand and TDC under the intervening mechanism of the quality of place. The findings of this study contribute to the research on urban TDC and provide new insights on urban tourism policy and sustainable destination management.

## Literature Review and Research Hypotheses

### Quality of Place

The quality of place refers to the characteristics of a place of different geographical scales that affect the quality of life of the inhabitants and the quality of experience of current and future visitors (Burton, 2014). The quality of place, thus, has two dimensions for fulfilling the expectations and needs of local residents and visiting tourists. Florida (2005) argued that the quality of place has three main components: rich amenities, unique or diverse lifestyles, and a superior natural and built environment. Regional differences in the combination of amenities can reflect differences in the preferences, lifestyles, and values of residents. Amenities are also physical objects that can be observed. Therefore, amenities have become the main focus of studies on the quality of place.

The concept of amenities comes from urban economics. It refers to the environment, events, facilities, behaviors, and services that make people feel happy and comfortable in a particular place (Wang, 2014). Early definitions of amenities were limited to nonproduced public goods without a clear price, such as the quality of climate (Gyourko and Tracy, 1991). Recent studies have divided amenities into urban and natural amenities. In the case of cities, amenities mainly refer to lifestyle amenities, which include both public services provided by the government, such as education and public safety (Gyourko and Tracy, 1991), and consumption-related businesses (Silver and Clark, 2016). According to Glaeser et al. (2001), there are four kinds of urban amenities: “a rich variety of services and consumer goods,” “aesthetics and physical setting,” “good public services,” and “speed” (p. 28). On the contrary, people in the countryside or natural areas place more emphasis on clean air, pleasant weather, clean water, forests, and other nature-related factors (Knapp and Graves, 1989; Rudzitis, 1999), which are called natural amenities by Silver and Clark (2016). Thus, compared with natural amenities, urban amenities mainly refer to the facilities and services provided by the public and private sectors. They are products that can be produced by human activities.

If the geographic scale of the study subject is narrowed from cities or regions to communities, studies on the quality of place then focus on “scenescapes.” A scene is a combination of people, activities, architecture, environment, and values that reflect the lifestyle of a community and the cultural or esthetics characteristics of a place (Silver and Clark, 2016). Florida (2005) asked three questions about the quality of place: What is there (amenities)?, Who is there?, and What happens there (activities)? The theory of scene involves answering questions from the community scale. Therefore, the theory of scene is consistent with the theory of the quality of place. But, there are also differences between the two theories. The theory of scene argues that there are only differences but no distinction between high and low among the lifestyles and values conveyed by scenes. Therefore, the measurement of scenes is a measure of cultural differences in communities. However, the measurement of the quality of place is based on the values and lifestyles upheld by the creative class, so areas with a high quality of place are favored by the creative class.

### Quality of Place and Urban Tourism

Previous studies discussed the relationship between the quality of place and tourism from two aspects: scenes and amenities. First, the lifestyle carried by the scenes is the core attraction of cultural tourism. Participants or observers of scenes are also known as tourists (Silver and Clark, 2016), and tourists immerse in their authentic and unique experience in the scenes (MacCannell, 1976; Benson and O’Reilly, 2009). Second, amenities such as landscape quality, theaters, parks, museums, restaurants, and shopping opportunities can be psychologically appealing to tourists (Johnson and Beale, 1994), which is also due to the lifestyle represented by the amenities. By consuming amenities, individuals maintain their lifestyles that can reflect their identities (Beck, 1992; Bauman, 2000). The tourists and long-term inhabitants motivated by the consumption of amenities are recognized as amenity migrants (Moss, 2006). Cities full of amenities have been turned into “festival cities” and “entertainment machines” (Clark, 2011; Wise, 2016). These cities attract not only the creative class, but also tourists.

In summary, tourists are experiencing scenes and consuming amenities in an unusual environment. Therefore, the unique quality of place of each region is “a competitive asset for attracting tourists” (Reilly and Renski, 2008, p. 17). The quality of place affects the destination choices of tourists.

### Tourism Destination Competitiveness

Though defined differently by TDC researchers due to the complexity and variations of resource endowments in destinations, the levels of development and governance, factors of market and industry supply, and the support of local residents, Cracolici and Nijkamp (2008) articulated that TDC is determined by the ability of the destination to provide tourists with a highly memorable and personalized travel experience. Therefore, TDC refers to the ability to provide products and services that meet and satisfy the growing and changing demand of tourists (Hassan, 2000; Ritchie and Crouch, 2000), thus improving and gaining market share (D’Hauteserre, 2000; Enright and Newton, 2004). According to Porter (1990), competitiveness is influenced by local basic conditions such as production factors, local demand, related and supporting industries, and market structure. Therefore, market share is affected by the supply capacity of products and services, which is subsequently affected by the local basic conditions of the destination, i.e., there is a cause-and-effect relationship between these three. As a result, TDC can be directly measured by market share or indirectly measured by products and services or basic conditions provided by the destination.

In terms of measuring TDC, some studies directly used demand-side indicators such as the Top 100 City Destinations Ranking (Geerts and Euromonitor International, 2017) and Mastercard’s Global Destination Cities Index (Hedrick-Wong et al., 2016). Other studies used basic conditions of the destination as factors and related industries to construct the index system (Crouch and Ritchie, 1999). Still, others have mixed the above three aspects to construct indicator systems of TDC such as Dwyer and Kim (2003), Zhang et al. (2011), and the Annual Report on the Development of World Tourism Cities (Wöber and World Tourism Cities Federation, 2018). However, Mendola and Volo (2017) argued that this kind of measurement does not distinguish the cause and effect relationship between demand, products, and factors and, thus, affects the quality of the indicators.

Assaker et al. (2013) conducted a path mechanism analysis on TDC of 154 countries and tested the causal relationship between economy and TDC under the intervening mechanism of environment and infrastructure. In this study, they tested country samples using economic indicators as a predictor, environmental indicators, and infrastructure indicators as mediators, and international tourist arrivals and international tourist receipts as an outcome. The study found that the economy had a positive indirect effect on TDC through the mediation of the natural environment and infrastructure. Furthermore, the natural environment and infrastructure had a positive direct effect on TDC. Therefore, the economy is a fundamental factor that influences TDC. The significance of this research is to point out a new direction for TDC research, namely to explore and understand the formation mechanism of TDC.

### Research Hypotheses

The goal of this study was to test the relationship between local demand and urban TDC using the quality of place as a mediator. Following basic conditions–product–demand modeling approach by Assaker et al. (2013), this study treated local demand as a basic condition, viewed the quality of place as a tourism attraction product, and then explored the quality of place as the intervening mechanism between local demand and urban TDC. Population and gross domestic product (GDP) per capita were used to describe the size and level of local demand. GDP per capita reflects the degree of urban wealth. The residents of wealthy cities have higher requirements for the quality of place and wealthy cities can invest in urban amenities with high quality, so there is a positive relationship between GDP per capita and quality of place. Population reflects the size of the local demand. Faber and Gaubert (2019) introduced population in the analysis of tourism to discuss the relationship between population and destination development. The relationship between population and the quality of place is complicated, as it may be considered a double-edged sword. Faber and Gaubert (2019) argued that the concentration of the population may enrich local entertainment activities, bring better urban amenities, and, thus, increase urban attractiveness, but it can also cause such “urban diseases” as congestion, as Glaeser (2011) discussed in *Triumph of the City*:

Cities attract poor people…. It is hard to miss the costs of concentrated poverty. Proximity makes it easier to exchange ideas or goods, but also easier to exchange bacteria or purloin a purse. All the older cities of the world have suffered the great scourges of urban life: disease, crime, and congestion (p. 9).

For small- and medium-sized cities, the main question is whether the population of the city can support the fixed cost of large urban amenities. For large cities, the decline in the quality of place brought by the increase in population is the focus of attention. Since this study used global cities as research samples, the population is assumed to have a negative impact on the quality of place.

Urban TDC is measured by international overnight visitors and per capita international overnight visitor spend, obtained from Mastercard’s Global Destination Cities Index (Hedrick-Wong et al., 2016). According to Reilly and Renski (2008), a positive relationship is assumed between the quality of place and urban tourism. The higher the quantity, quality, and diversity of the amenities are, the stronger the attractiveness of the tourist destination will be.

Based on the above literature review and conceptual underpinnings, we formulated four hypotheses involving the quality of place. GDP per capita has a positive effect on the quality of place, the population has a negative effect on the quality of place, the quality of place has a positive effect on per capita international overnight visitor spend, and the quality of place has a positive effect on international overnight visitors. Therefore, we propose four hypotheses to test the intervening mechanism of the quality of place between local demand as urban economy and TDC:

*Hypothesis 1.* The positive indirect relationship between local GDP per capita and per capita international overnight visitor spend is mediated by the quality of place.
*Hypothesis 2.* The negative indirect relationship between local population and per capita international overnight visitor spend is mediated by the quality of place.
*Hypothesis 3.* The positive indirect relationship between local GDP per capita and international overnight visitors is mediated by the quality of place.
*Hypothesis 4.* The negative indirect relationship between the local population and international overnight visitors is mediated by the quality of place.

Moreover, it is assumed that the size and level of local demand has a positive direct effect on international overnight visitors because there are more businesses and activities in larger and wealthier cities and thus these cities can attract more visitors. The size and wealth of cities are also assumed to have a positive and direct effect on per capita international overnight visitor spend, as the price of production factors such as land and wages is much higher in larger and wealthier cities, therefore tourists have to pay higher fees for hotels, restaurants, and city transportation in these cities. We present the following four hypotheses to test the relationships between local demand and urban TDC.

*Hypothesis 5.* Local GDP per capita has a significant positive direct impact on per capita international overnight visitor spend.
*Hypothesis 6.* The local population has a significant positive direct impact on per capita international overnight visitor spend.
*Hypothesis 7.* Local GDP per capita has a significant positive direct impact on international overnight visitors.
*Hypothesis 8.* The local population has a significant positive direct impact on international overnight visitors.

**FIGURE 1:**
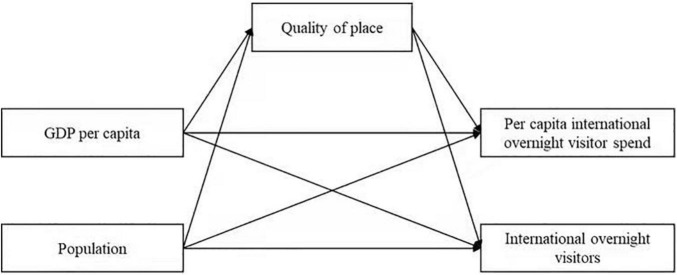
“Local demand-quality of place-urban tourism” mediation model.

Figure 1 presents the local demand–the quality of place–urban tourism mediation model of the study, highlighting the factors and their proposed relationships.

## Materials and Methods

### Measurement of the Quality of Place

The study selected 50 cities ranked above the “alpha” level in the Globalization and World Cities Research Network city rankings as samples and measured the quality of place of these global cities. Florida (2003) suggested that the indicator system of the quality of place should include three dimensions: technology, talent, and tolerance (“3T”). Trip (2007) opined for “creativity and talent,” “diversity, tolerance, and safety,” and “specific amenities” in his study of Netherlands and assigned equal weight to all the indicators. Trip’s indicator system is a combination of measurement of the quality of place by Florida (2003) and measurement of amenities by Glaeser et al. (2001).

Considering the data availability and the study purpose of measuring 50 global cities across the world, this study adopted and modified Trip’s (2007) indicators. First, in the “creativity and talent” dimension, the proportion of PC possession and the proportion of population aged 15+ years with higher education were used to reflect the size of the creative class. Glaeser (2005) argued that creativity is derived from skilled people with high education. The gathering of talent can bring new ideas for creative endeavors. Therefore, this study used the above two indicators to measure the “creativity and talent” dimension.

Second, the types of cuisines, the proportion of international residents, and the safety index were used to measure the dimension of “diversity, tolerance, and safety.” The cuisine is an important part of the culture that is influenced by natural factors such as climate and geography and human factors such as social status and religious beliefs. Therefore, food can express the identity of consumers (Montanari and Sonnenfeld, 2006). Understanding the diversity of food is beneficial to understand the diversity of culture. Silver and Clark (2016, p. 111) also believed that the diversity of culture could be reflected through cuisines. Compared with cuisines, the proportion of international residents and the safety index are more intuitive reflections of the inclusiveness and safety of the city, which have been used in previous studies, such as Trip’s study in 2007.

Third, in the dimension of “specific amenities,” this study did not consider the distance from cities to natural areas, waters, and oceans, but only considered the cultural and sporting activities, recreational facilities, and restaurants in cities. This is because the natural environment is more related to the quality of place of rural areas (Knapp and Graves, 1989; Rudzitis, 1999), while the urban quality of place focuses on lifestyle amenities (Reilly and Renski, 2008), namely, culture and public services.

Finally, this study added the “environment” dimension. As Florida (2005) noted, the environment is an important component of the quality of place. We analyzed several environmental pollution indexes for the sample cities, namely, air, water, noise, and light pollution measures, and also added city cleanliness indicators for the environmental dimension. To compare values from different datasets, this study normalized all the data between 0 and 1 and assigned them equal weight.

The data required for the quality of place measurement came from the Euromonitor Database, Numbeo Database, and TripAdvisor. TripAdvisor is a travel review business website that contains over 500 million reviews and covers cities in more than 190 countries. The study used Python 3.6 to crawl the data of 50 global cities on TripAdvisor. The specific indicator system and data sources of the quality of place are shown in Table 1.

**TABLE 1:** The indicator system and data sources.

Indicators		Indicator Interpretation	Data sources
Creativity and talent	PC possession	Percentage of households with personal computers	Euromonitor
	Education	Percentage of population aged 15+ with higher education	Euromonitor
Diversity, tolerance, and safety	Cuisine	Types of cuisine per thousand people	TripAdvisor, Euromonitor
	Foreign citizens	Number of foreign citizens per thousand people	Euromonitor
	Safety	The crime index	Numbeo
		The public security and political stability index	Institute for Economics and Peace
Amenities	Typical culture amenities	Total number of museums	List of most visited famous museums provided by Wikipedia
		Total number of world-famous opera houses	Opera house list provided by Wikipedia
		Number of performances per thousand people	TripAdvisor, Euromonitor
	Events	Total number of international sport events	World major sport events from mapsofworld.com
		Total number of world-famous music festivals	World famous music festivals compiled by Time Out Magzine
		Total number of international film festivals	International film festivals certified by FIAPF
	Recreation	Number of tourist attractions per thousand people	TripAdvisor, Euromonitor
		Ratio of public green area to urban area	Easypark
	Catering	Number of catering per thousand people	TripAdvisor, Euromonitor
Environment	Environmental pollution	Air pollution index	Numbeo
		Water pollution index	Numbeo
		Noise and light pollution index	Numbeo
	City cleanliness	Dirty and untidy index	Numbeo

### Mediation Model

Based on the model hypotheses and Hayes (2013), the study proposed the following indirect effect model:


$$\begin{array}{c} \mathrm{Quality}\mathrm{of}\mathrm{place}\\ \;=\alpha_{1}+\beta_{11}\times \mathrm{GDP}\mathrm{per}\mathrm{capita}+\beta_{12}\times \text{Population}+\varepsilon_{1} \end{array}$$

$$\begin{array}{c} \mathrm{Per capita international overnight visitor spend}\\ \;=\alpha_{2}+\beta_{21}\times \mathrm{GDP}\mathrm{per}\mathrm{capita}+\beta_{22}\times \text{Population}+\beta_{23}\\ \;\times \mathrm{Quality}\mathrm{of}\mathrm{place}+\varepsilon_{2} \end{array}$$

$$\begin{array}{c} \mathrm{International}\mathrm{overnight}\mathrm{visitors}\\ \;=\alpha_{3}+\beta_{31}\times \mathrm{GDP}\mathrm{per}\mathrm{capita}+\beta_{32}\times \text{Population}+\beta_{33}\\ \;\times \mathrm{Quality}\mathrm{of}\mathrm{place}+\varepsilon_{3} \end{array}$$

Hayes (2013) provided a plug-in in SPSS called PROCESS to test the mediating effect by bootstrapped CI. Bootstrapping estimates the nonnormal sampling distribution of the index of mediation and generates a CI after random sampling from the original sample with a replacement. Thus, the bootstrapping method is suitable for nonnormal distributed samples. For 90% CI, if the CI does not straddle zero, the mediating effect is supported.

Table 2 presents the descriptive statistics of variables used for the analysis. GDP per capita and population were collected from Passport Economics: Cities (Euromonitor, 2018). Per capita international overnight visitor spend and international overnight visitors were from the Global Destination Cities Index by Mastercard (2018).

**TABLE 2:** Descriptive statistics of variables.

Variable	*N*	Mean	Std. Dev.	Min	Max
GDP per capita (thousand, $)	50	41.15	28.80	4.41	109.95
Population (million)	50	11.22	8.89	1.52	37.48
Per capita international overnight visitor spend ($)	49	939.24	415.34	368.19	2049.77
International overnight visitors (million)	49	6.12	5.00	0.99	21.47
Quality of place	50	0.51	0.17	0.13	0.78

*Sources: Euromonitor, 2018; Mastercard, 2018.*

## Results

### Measurement of the Quality of Place

**FIGURE 2:**
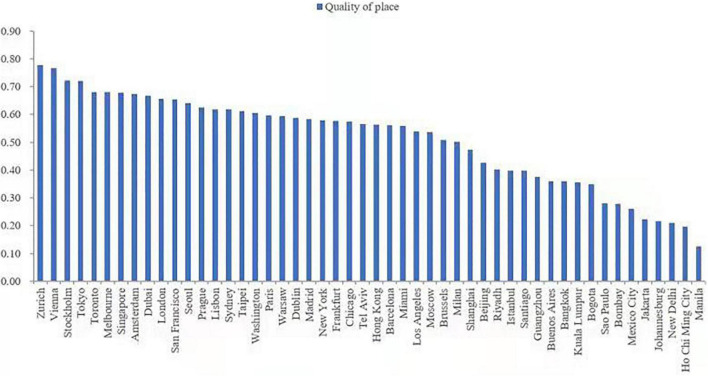
Results for quality of place measurement.

As shown in Figure 2, cities with the high quality of place, such as Zurich, Vienna, and Stockholm, were mostly those in developed countries, while cities with the low quality of place, such as New Delhi, Ho Chi Minh City, and Manila, were mainly located in developing countries. Among the 31 cities with the quality of place above average (0.51), 15 were in Europe, 7 in North America, 7 in Asia, and 2 in Oceania. The 19 cities with the lower quality of place were located in Asia, South America, and Africa, except Milan.

**FIGURE 3:**
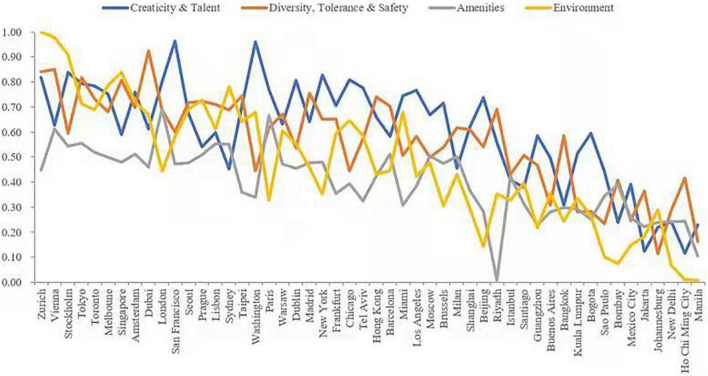
Result for the four-dimensional measurement of quality of place.

Considering all the four dimensions of the quality of place (Figure 3), cities in developing countries had smaller gaps with those in developed countries in the dimension of “creativity and talent.” For example, Beijing and Shanghai exceeded the average in this dimension. Bogota, Guangzhou, Riyadh, Kuala Lumpur, and Buenos Aires had higher scores than some developed cities such as Milan and Sydney. However, in terms of “diversity, tolerance, and safety” and “amenities,” developing cities lagged behind developed cities. The safety index led to lower rankings for United States cities such as Los Angeles, Miami, Chicago, and Washington, DC. In terms of “environment,” cities in developing countries still lagged behind those in developed countries, but the scores of large cities such as London, New York, Paris, and Los Angeles were also below the average in the environmental dimension.

### Test of Mediation

As one of the main goals of this study is to examine the quality of place as an intervening mechanism between local demand and TDC, we needed to perform a mediation analysis to analyze the indirect effect between the predictors and the outcome. For this study, we did not follow the three-step procedure recommended by Baron and Kenny (1986) since we could perform the mediation analysis using two regression models (Hayes, 2013). From a methodological point of view, it has been also suggested that it is not necessary to test the total effect of the independent variable on the dependent variable before testing the mediating effect (MacKinnon et al., 2000; Zhao et al., 2010). If there is a significant correlation between the independent variable and the dependent variable, the next step in the study is to examine whether there is a mediating effect between these two variables—whether the independent variable will affect the dependent variable through the mediator variable (Baron and Kenny, 1986). If there is no significant correlation between the variables, this study can consider whether there is a suppression effect in the model, that is, the opposite sign of the direct and indirect effect makes the total effect obscured (MacKinnon et al., 2000; MacKinnon, 2008). Therefore, the test of mediating effects can continue even when dependent variables do not correlate with independent variables.

Based on the hypotheses, the quality of place played a mediating role between local demand and urban tourism competitiveness. We applied Model 4 from PROCESS 3.3 (Hayes, 2013), with GDP per capita or population as the independent variable, the other one as the covariate, the quality of place as the mediator variable, and per capita international overnight visitor spend and international overnight visitors, respectively, as the dependent variables. Then, we tested the mediating effect by using a 5,000-bootstrap sample under the 90% CI.

As shown in Table 3, GDP per capita had a significant positive impact on the quality of place (0.462, *p* < 0.001), while the population had a significant negative impact on the quality of place (−0.133, *p* = 0.024). In terms of per capita international overnight visitor spend, the indirect effect of GDP per capita or population via the quality of place on urban tourism was not significant (bootstrapped CI, −0.325, 0.341; −0.092, 0.094). Therefore, hypothesis 1 and hypothesis 2 were not supported. Test results showed that per capita international overnight visitor spend was directly influenced by the two indicators of local demand (0.600, *p* = 0.006; 0.306, *p* = 0.032), thus supporting hypothesis 5 and hypothesis 6. However, in terms of overnight international visitors, the relationships between GDP per capita and international overnight visitors, and between population and international overnight visitors, were mediated by the quality of place (bootstrapped CI, 0.131, 0.745; −0.296, −0.011). Hypothesis 3 and hypothesis 4 were verified. The higher the quality of place, the more international overnight visitors there were. The relationship between these two was significant (0.868, *p* = 0.020). The number of international overnight visitors was also directly affected by population (0.409, *p* = 0.007), so the mediating role of the quality of place was partial. In addition, hypothesis 7 and hypothesis 8 were also verified as the relationships between GDP per capita and international overnight visitors, and between population and international overnight visitors were both significantly positive.

**TABLE 3:** Results for mediation model.

	Coeff	Se	*t*	*p*	LLCI	ULCI

Outcome variable: quality of place						
Constant	0.328	0.054	6.089	0.000	0.220	0.437
GDP per capita	0.462	0.058	8.018	0.000	0.346	0.579
Population	−0.133	0.057	−2.343	0.024	−0.246	−0.019
*R*^2^ = 0.707, *F* = 55.577***						

**Outcome variable: per capita overnight international visitor spend**						

Constant	−0.122	0.167	−0.728	0.470	−0.458	0.215
GDP per capita	0.600	0.206	2.911	0.006	0.185	1.016
Quality of place	−0.052	0.340	−0.152	0.880	−0.737	0.634
Population	0.306	0.138	2.215	0.032	0.028	0.584
*R*^2^ = 0.296, *F* = 6.313***						

**Outcome variable: overnight international visitors**						

Constant	−0.328	0.176	−1.862	0.069	−0.682	0.027
GDP per capita	−0.145	0.217	−0.666	0.509	−0.582	0.293
Quality of place	0.868	0.358	2.423	0.020	0.146	1.590
Population	0.409	0.145	2.813	0.007	0.116	0.702
*R*^2^ = 0.201, *F* = 3.781***						

**Indirect effect: GDP per capita as the independent variable, per capita overnight international visitor spend as the dependent variable**						

	**Effect**	**BootSE**	**BootLLCI**	**BootULCI**		

Quality of place	−0.024	0.169	−0.325	0.341		

**Indirect effect: population as the independent variable, per capita overnight international visitor spend as the dependent variable**						

	**Effect**	**BootSE**	**BootLLCI**	**BootULCI**		

Quality of place	0.007	0.058	−0.092	0.094		

**Indirect effect: GDP per capita as the independent variable, overnight international visitors as the dependent variable**						

	**Effect**	**BootSE**	**BootLLCI**	**BootULCI**		

Quality of place	0.401	0.155	0.131	0.745		

**Indirect effect: population as the independent variable, overnight international visitors as the dependent variable**						

	**Effect**	**BootSE**	**BootLLCI**	**BootULCI**		

Quality of place	−0.115	0.093	−0.296	−0.011		

***p < 0.01.

## Discussion

Using the quality of place as an intervening mechanism between local demand, measured by GDP per capita and urban population, and urban TDC, measured by international overnight visitors and international overnight visitor spend, this study found two revealing and significant results. First, the local demand level, namely, urban wealth, is positively related to the competitiveness of urban tourism. Second, local demand, namely, urban population, has both positive and negative effects on urban TDC. In the traditional experience, the influence of urban wealth and city size on urban TDC has not been clearly understood. The mediating role of the quality of place between local demand, e.g., urban wealth and urban population, and urban tourism competitiveness sheds new light on these relationships.

As demonstrated in the model test, the quality of place plays a mediating role in the relationship between local demand and urban TDC. When it comes to the relationship between GDP per capita and international overnight visitors, the quality of place has a full mediating effect between the two variables. The role of the quality of place as a mediator variable helps understand the logical relationship between local per capita income and overnight visitors. Rich cities are able to invest more in building and improving the quality of place amenities and the higher quality of place in rich cities will, in turn, attract more tourists. Local demand level is an important factor in the competitiveness of urban tourism. This is in line with Porter’s (1990) theory that if the local market is the leading market, then it tends to have a competitive advantage.

The study did not find an association between the quality of place and per capita overnight international visitor spend. While the urban size and urban affluence have a positive and direct effect on per capita tourism spending—i.e, tourists spend more in larger cities and wealthier cities—it was not supported that the higher quality of place leads tourists to spend more. One possible explanation is that many urban amenities and urban scenes that reflect lifestyles are free, such as parks, museums, historical and cultural heritage sites, urban trails, street art, and shop window displays, which are paid for by residents in the form of taxes. Urban amenities and urban scenes are typically noncompetitive and nonexclusive public goods. It is difficult for city and community managers to charge tourists. At the same time, urban amenities and urban scenes that reflect local culture are favored by tourists as authentic tourism products. Therefore, after introducing the quality of place, we came to a revealing conclusion: high-quality urban amenities and urban scenes increase tourists, but the free access to them does not increase per capita spending of tourists. This can explain the results of this study that the mediating effect of the quality of place is positive and significant between GDP per capita and international overnight visitors, but not significant between GDP per capita and per capita international overnight visitor spend.

Although the quality of place does not necessarily increase per capita spending of tourists, investing in the quality of place is still an effective way for local governments to promote the prosperity of the tourism and business sectors because the quality of place attracts tourists and this will increase the income of the tourism and commercial enterprises. Investing in the quality of place has spillover effects on the local consumption sectors, as evidenced by the positive and direct effect between GDP per capita and per capita international overnight visitor spend.

We found two opposite influence paths of urban population on international overnight visitors. Larger population and higher population densities increase urban productivity. Higher productivity, as a result, will attract large influxes of people to search for opportunities in the city, which leads to “urban diseases” such as crime, congestion, and environmental pollution (Glaeser, 2011). The larger the population is, then the lower or more imbalanced the quality of place will be. Through the mediation of the quality of place, urban population and international overnight visitors are negatively correlated. However, a large population brings a city social vitality and cultural diversity, and the city is more likely to be open and economically connected with the outside world, which will increase the diversity of urban tourist attractions. Meanwhile, a large population is also beneficial for sharing the high fixed cost required for large urban amenities that attract tourists. In our study, there was a positive association between population and international overnight visitors, with a correlation coefficient of 0.287 (*p* < 0.05). However, this positive direct effect suppressed the negative indirect relationship between population and overnight visitors through the mediation of the quality of place, therefore, a “suppression effect” was detected (MacKinnon et al., 2000; Shrout and Bolger, 2002). The mediating effect of the quality of place helped uncover this indirect negative effect of the population.

The indirect negative effect of the quality of place found in this study can enable us to gain a deeper understanding of the relationship between city size and urban TDC. Urban size improves urban TDC, as a large population benefits cultural diversity, economic vitality, and social tolerance. On the other hand, city size undermines the competitiveness of urban tourism because of the “urban diseases” it causes. How to expand the positive effect and mitigate the negative effect is a challenge for policymakers.

## Implications and Future Research

This study emphasizes the important role of the quality of place in urban TDC and points out how the quality of place mediates the relationship between local demand and urban tourism competitiveness. The findings of the study contribute to both the theory and practice. Both the theoretical and practical implications are discussed in the following sections.

### Theoretical Implications

In terms of theoretical contribution, this study adds to the new stream of studies (Assaker et al., 2013) by examining the intervening mechanism between the predictors of macroeconomic factors and TDC. Prior studies on the relationships of antecedents and TDC were mainly conducted by analyzing direct links between the independent predictors and outcome variables. By introducing the mediating variable of the quality of place, this study finds that local market demand is a factor of urban TDC. Through the empirical study on urban TDC, we have verified an important point in theory by Porter that local demand affects the international competitiveness of an industry. Our research confirms that the local market has an impact on the international competitiveness of urban tourism destinations. This is a significant theoretical contribution of this research.

We have also discovered two effects of city size on urban TDC. First, the scale of the city has a positive effect on urban TDC. This is in line with the theory of spatial economics on local market effects. However, by introducing the quality of place as a mediator, we uncovered an indirect negative effect of urban population on urban TDC, i.e., the larger the urban population, the more prominent the problem of urban diseases, and the lower the quality of place, which in turn causes urban population to have a negative impact on urban TDC. These are two opposite mechanisms of action. By introducing the mediation variable of the quality of place, we can clearly distinguish these two mechanisms of action. This is the second theoretical contribution of this research.

More specifically, this study integrates the concept of the quality of place in the model testing for TDC studies. The quality of place is an important aspect of a destination that appeals to both residents and tourists. Therefore, the study contributes to the literature by considering the quality of place when determining TDC. Local demand size has both positive and negative effects on urban TDC. The positive effect is expressed as a larger market size attracting more visitors, which can be explained by the spatial interaction theory. The negative effect is expressed as a larger local population causing the lower quality of place and subsequently reducing visitors. The negative effect is consistent with the view of Faber and Gaubert (2019).

Our findings enrich the literature on tourism destination competitiveness by elucidating the role of the quality of place in urban economic development, not just for attracting the creative class (Florida, 2002, 2005) but also for enhancing urban tourism competitiveness. Furthermore, the quality of place as a mediator enables researchers to uncover the suppression effect between local demand and tourism destination competitiveness. Without introducing the quality of place, we could not detect this negative relationship, because the positive direct effect masks the negative indirect effect, that is, the existence of “suppressing effects” (MacKinnon, 2008). The mediated test of TDC can provide nuanced insight into destinations competing for tourism business by recognizing the critical role of the quality of place and teasing out the suppression effect on the relationship.

### Practical Implications

Practically, this study provides implications for urban planning, tourism policies, and destination management. First, the quality of place can attract not only the creative class but also tourists, so it is necessary to invest in the quality of place to build a city shared by both hosts and guests. Therefore, local governments should not only invest in scenic attractions that directly accommodate tourists, but also invest in the quality of place, which is an important way to promote urban tourism development, i.e., local governments should actively invest in urban amenities and create a more tolerant, diversified, inclusive, and safe social environment to attract the creative class and tourists. Second, as it is reported in the findings, the population size has two effects on the size of overnight visitors: the positive direct effect and the negative indirect effect by the mediator of the quality of place, which implies that large population leads to declining quality of place, such as congestion, pollution, and safety issues, and declining quality of place reduces tourist visits. In this case, urban destination managers should coordinate with the municipal government and stakeholders to mitigate the negative aspects of the quality of place and improve the quality of place for a memorable and safe travel experience. Third, urban destinations, particularly cities facing aging infrastructure and amenities, need to stay competitive by ongoing investments in upgrading and maintaining the quality of place. The new wave of competition is to develop smart destinations for the digitalization of travel information and service satisfaction (Koo et al., 2016).

### Limitations and Future Research

Though this study has plowed the fertile field of TDC from a new perspective by testing the quality of place as the mediator, it has its limitation and also opens new areas for future academic inquiry. The measurement of the quality of place needs to consider a myriad of factors essential for different cities. This study attempted to be comprehensive in modeling the four dimensions of the quality of place, but the uniformity and adequacy of the quality of place measures need to be reviewed for future studies. Though we included pollution and cleanliness measures for the sample cities, the range of qualities may include other aspects, such as the aesthetics of the cityscape (Andrews, 2001). Future studies should continue to refine and improve the model of the quality of place in TDC studies. New research can also consider the different sizes of cities, such as large, midsize, and small cities, and determine how the quality of place mediates the relationship between local demand and TDC (Kelly et al., 2017). Since the attributes for the quality of place encompass mostly physical, technological, and safety factors, smaller cities or towns may win competitiveness by authenticity and individual charm.

## Data Availability Statement

Publicly available datasets were analyzed in this study. All data sources were listed in Table 1. Data for quality of place measurement were collected from the Euromonitor Database, https://www.euromonitor.com/our-expertise/passport, Numbeo Database, https://www.numbeo.com/quality-of-life/, and TripAdvisor, http://www.tripadvisor.com/.

## Author Contributions

JW, JX, and LY contributed to conception and design of the study. JW organized the database. JX performed the statistical analysis. JW and JX wrote the first draft of the manuscript. LY wrote sections of the manuscript. All authors contributed to manuscript revision, read, and approved the submitted version.

## Conflict of Interest

The authors declare that the research was conducted in the absence of any commercial or financial relationships that could be construed as a potential conflict of interest.

## Publisher’s Note

All claims expressed in this article are solely those of the authors and do not necessarily represent those of their affiliated organizations, or those of the publisher, the editors and the reviewers. Any product that may be evaluated in this article, or claim that may be made by its manufacturer, is not guaranteed or endorsed by the publisher.
